# Pooled Antibiotic Susceptibility Testing for Polymicrobial UTI Performs Within CLSI Validation Standards

**DOI:** 10.3390/antibiotics14020143

**Published:** 2025-02-01

**Authors:** Richard A. Festa, Frank R. Cockerill, Rick L. Pesano, Emery Haley, Natalie Luke, Mohit Mathur, Xiaofei Chen, Jim Havrilla, Michael Percaccio, Jesus Magallon, Shane Erickson, Mandana Ghashghaie, Alain Rosas, David Baunoch

**Affiliations:** 1 Department of Research and Development, Pathnostics, Irvine, CA 92618, USA; rfesta@pathnostics.com (R.A.F.); mpercaccio@pathnostics.com (M.P.); jmagallon@pathnostics.com (J.M.); serickson@pathnostics.com (S.E.); mghashghaie@pathnostics.com (M.G.); arosas@pathnostics.com (A.R.); 2 Partner, Trusted Health Advisors, Orange, CA 92675, USA; frankcockerill@trustedhealthadvisors.us (F.R.C.); rickpesano@trustedhealthadvisors.us (R.L.P.); 3 Department of Clinical Research, Pathnostics, Irvine, CA 92618, USA; ehaley@pathnostics.com (E.H.); nluke@pathnostics.com (N.L.); 4 Department of Medical Affairs, Pathnostics, Irvine, CA 92618, USA; mmathur@pathnostics.com; 5 Department of Data and AI, Pathnostics, Irvine, CA 92618, USA; xchen@pathnostics.com (X.C.); jhavrilla@pathnostics.com (J.H.)

**Keywords:** urinary tract infection, polymicrobial, antibiotic susceptibility testing, antibiotic resistance, pooled antibiotic susceptibility testing, heteroresistance, disk diffusion, broth microdilution

## Abstract

Background/Objectives: Urinary tract infections (UTIs) pose an increasing risk of antimicrobial resistance, and novel diagnostic tests have been developed to address the limitations of standard urine culture in these cases. It is important that these novel tests be validated for agreement and error rates against the standard antibiotic susceptibility testing (AST) methods. Methods: Polymicrobial (≥two non-fastidious microorganisms) consecutive clinical urine specimens submitted for UTI diagnostic testing were included in this analysis. Specimens were tested with Pooled Antibiotic Susceptibility Testing (P-AST) and with broth microdilution/disk diffusion (BMD/DD) in parallel. Performance characteristics, such as essential agreement (EA%), very major errors (VMEs), and major errors (MEs), were assessed using Clinical and Laboratory Standards Institute (CLSI) standards. Specimens with P-AST-resistant and BMD/DD consensus-sensitive results were assessed for heteroresistance. Real-world clinical sample data were used to assess associations between increasing organism counts and average “sensitive” antibiotic count per sample. Results: The essential agreement between P-AST and standard isolate AST was ≥90%, VMEs were <2.0%, and MEs were <3.0%, meeting the CLSI guidelines for AST verification and validation studies. When heteroresistance was accounted for, overall VMEs and MEs were both <1.5%. The presence of additional non-fastidious organisms dropped the number of average “sensitive” antibiotics from 9.8 with one organism to 2.5 with five or more organisms. The presence of fastidious organisms did not have any meaningful impact. Conclusions: P-AST, a component of the Guidance^®^ UTI assay (Pathnostics, Irvine, CA, USA), performed within CLSI standards for AST in polymicrobial UTI diagnostic urine specimens.

## 1. Introduction

In 2019, over one-quarter million deaths globally were attributed to or complicated by antimicrobial-resistant urinary tract infections (UTIs) [1]. Therefore, antibiotic susceptibility testing (AST) is being increasingly relied on as an essential part of the work of clinical microbiology laboratories [2]. The current standard diagnostic test utilized for UTIs is the standard urine culture (SUC) with AST testing [3,4]. Due to known limitations of this technology, especially in polymicrobial infections, newer assays have been developed for complicated and recurrent UTIs [5,6,7,8,9]. These tests attempt to reduce the time to results, provide the ability to detect polymicrobial infections, and improve the detection of urine pathogens, including fastidious organisms [10,11,12,13]. As these assays are increasingly used in clinical practice, it is important to validate them against standard AST methods to evaluate error rates and agreement.

### 1.1. M-PCR/P-AST (Guidance UTI) Assay

One of the novel assays developed for complicated and recurrent UTIs is a combination of multiplex polymerase chain reaction (M-PCR) and Pooled Antibiotic Susceptibility testing (P-AST) [10,14,15,16,17]. The M-PCR component identifies and quantitates microorganisms and detects antimicrobial resistance genes [6,11,18,19,20,21]. Simultaneously, the P-AST component utilizes a fluorescent probe to rapidly measure the metabolic activity of living bacteria in the pooled urine organisms and reports antibiotic susceptibility according to the most restrictive CLSI minimum inhibitory concentration (MIC) breakpoints of all identified non-fastidious organisms (Supplemental Table S1 and Supplemental Figure S1). Since the entire pool of microorganisms in the specimen is exposed to antibiotics simultaneously, the MIC determined by P-AST reflects the collective susceptibility of the microbial community [5]. This incorporates the interactions and resistance dynamics of all detected species rather than assessing each species and/or strain in isolation. As a result, P-AST is designed to account for both heteroresistance and for effects of multi-species interactions that may alter susceptibility in polymicrobial infections [22]. The use of the assay that P-AST is a component of, Guidance^®^ UTI (Pathnostics, Irvine, CA, USA), has previously been associated with faster turnaround time, reduced empiric antibiotic therapy utilization, and improved clinical outcomes in patients with complicated or recurrent UTIs [10,15]. Guidance UTI is run out of Clinical Laboratory Improvement Amendments (CLIA)- or College of American Pathologists (CAP)-certified laboratories and has approval from the New York State Department of Health (NYDOH) Clinical Laboratory Evaluation Program (CLEP).

### 1.2. Standard Phenotypic Susceptibility Tests

Clinical microbiology labs frequently utilize automated systems, such as VITEK, Phoenix, and MicroScan systems, to perform phenotypic AST. However, these systems exhibit high result variability due to the variety of software versions and cards used [23]. Globally, the disk diffusion (DD) phenotypic AST method is the most widely used due to its low cost and relative ease [24]. However, DD AST is unable to determine quantitative MIC values, which are routinely used for conducting resistance surveillance, testing new antimicrobial agents, and performing validation of new AST methodologies and systems. Due to this limitation, the broth microdilution (BMD) method performed in 96-well microtiter plates is preferentially used as a standard comparator for validation of new AST assays [24]. Since all methods have significant variability, a common method of validation is to confirm the results of BMD with DD when called for.

### 1.3. Importance of Polymicrobial Infections in UTIs

Polymicrobial infections have been reported in up to 52% of suspected UTI cases in older adult populations [14,25,26,27] and have specifically been associated with poorer outcomes [28]. Polymicrobial co-cultures have repeatedly been found to have reduced antibiotic susceptibility relative to their monomicrobial counterparts. For example, antimicrobial resistance genes can be transferred between species via plasmids [29] or antibiotic-inducible prophages [30]. Additionally, collective antimicrobial tolerance/resistance phenomena enable multiple microbial species in a shared environment to work together to survive antimicrobial challenges. The three overarching mechanistic themes of collective antimicrobial tolerance/resistance are community-signaling-mediated upregulation of drug efflux pump expression [31], community-signaling-mediated growth modulation [32], and environmental modulation [33]. Environmental modulation can involve antimicrobial degradation enzymes such as β-lactamases [34], iron [35,36], ammonia [37] or elevated pH [38], and more. These interaction effects cannot be detected by phenotypic isolate AST [33,39,40,41,42,43,44,45,46,47]. In addition to cooperative mechanisms, simple co-infection by multiple species, each with its own intrinsic antibiotic resistance, can result in multi-drug- or pan-drug-resistant polymicrobial infection [48].

### 1.4. Limitations of Standard Urine Culture

Standard urine culture is generally limited in its ability to detect more than one or two organisms, leading to many cases of missed organisms or mixed flora/contamination results in cases of polymicrobial UTIs [3,10,11,12,15,18,25,49,50]. In addition, isolate AST testing does not account for the interactions detailed above when multiple pathogens are present together in the urinary tract. It also often fails to detect resistant subpopulations with low relative cell density compared to the sensitive subpopulation. Such populations have been called “heteroresistant” [51], a term coined to describe the phenomenon in which a subpopulation of bacteria has significantly lower susceptibility to an antibiotic than the main population [52]. Critically, the inability to efficiently detect and analyze heteroresistant bacterial populations has been posited to be both a driver of classical homogeneous resistance [53] and a cause of unexplained clinical antibiotic treatment failures [54,55].

### 1.5. Objective

**Figure 1:**
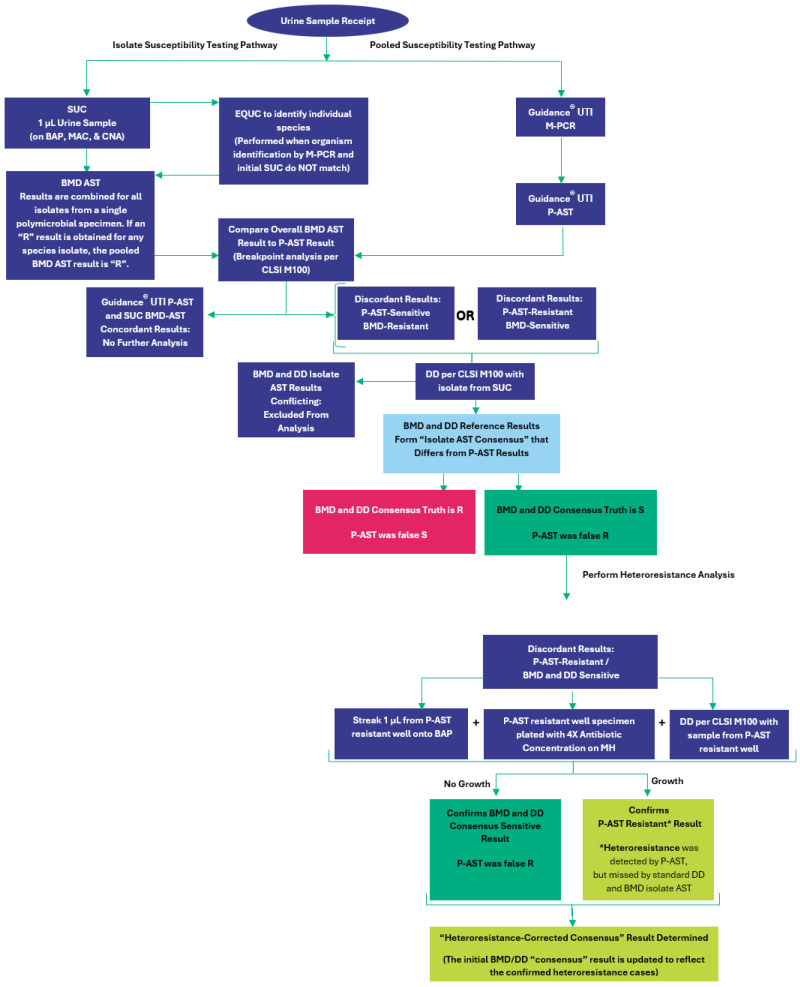
Overview of Study Workflow. SUC = standard urine culture; BAP = blood agar plate; EQUC = expanded quantitative urine culture; MAC = MacConkey agar plate; CNA = Columbia naladixic acid agar plate; BMD = microbroth dilution; DD = disk diffusion; AST = antibiotic susceptibility testing; CLSI = Clinical and Laboratory Standards Institute; MH = Mueller–Hinton agar; M-PCR = multiplex polymerase chain reaction; P-AST = pooled antibiotic susceptibility testing; Rz = resazurin.

Prior work has established the validity of P-AST against standard AST methods in monomicrobial clinical urine specimens [56]. This study evaluates the validity of P-AST in polymicrobial clinical urine specimens from UTI patients using the standards established by CLSI. We measure error rates and agreement when comparing results from the P-AST assay to standard methods of BMD and DD from the same urine specimen. This was analyzed both with and without correcting for heteroresistance (Figure 1). We also analyzed the impact of additional non-fastidious and fastidious organisms on the average number of susceptible antibiotics reported per case.

## 2. Results

The 193 polymicrobial specimens in this analysis were consecutive specimens that had ≥2 non-fastidious organisms detected. Of these, most specimens (79%) had just two non-fastidious organisms, 15% had three non-fastidious organisms, and only a few (6%) had four or more non-fastidious organisms present. When both fastidious and non-fastidious organisms were counted, 46% had exactly two organisms, 23% had three, 20% had four, and 11% had five or more (Table 1). A table of the relative frequencies of individual bacterial species/groups detected in the specimens included in this analysis can be found in Supplemental Table S2.

Based on this distribution, we performed an analysis of all polymicrobial specimens (≥2 non-fastidious organisms) as well as subset analyses for specimens with exactly two non-fastidious organisms (*n* = 153) and specimens with three or more non-fastidious organisms (*n* = 40).

### 2.1. Comparison of P-AST to Standard AST Using CLSI Criteria for Agreement and Errors

Urine specimens from 193 subjects were found to have two or more non-fastidious organisms with or without fastidious organism(s). The subjects who provided these specimens ranged in age from 30.8 to 100.0 years (median = 75.5) with an average age of 74.7 years (SD = 10.1). The subjects were 70% female (*n* = 135) and 30% male (*n* = 58). Each specimen was submitted with at least one International Classification of Diseases, Tenth revision, Clinical Modification (ICD-10-CM) code associated with a suspected UTI diagnosis and some specimens were submitted with multiple relevant ICD-10-CM codes (36 had two ICD-10-CM codes, four had three ICD-10-CM codes, one had four ICD-10-CM codes, and one had five ICD-10-CM). The most common of these codes are outlined in Table 2 Part A.

Of these 193 total specimens, 153 had exactly two non-fastidious organisms and 40 had three or more (Table 1). They had comparable demographics and ICD-10-CM codes as shown in Table 2 Part B and C.

Testing all 193 specimens against up to 19 antibiotics (organism dependent) resulted in 3550 organism–antibiotic combinations for comparison of both antibiotic susceptibility test method results (Table 3).

Contingency tables for analysis by microorganism subset can be found in Supplemental Tables S3–S6. P-AST performance metrics for all specimens and for subsets based on Gram staining or number of organisms are shown in Table 4. The Gram-positive and Gram-negative subsets included cases where all detected non-fastidious organisms had the same staining result and excluded cases where the organisms had different Gram stains.

The results were essential agreement > 90%, VMEs < 2%, and MEs < 3%, meeting CLSI criteria in all cases. Minor errors are allowed by CLSI, especially when most are minor errors with essential agreement. The categorical agreement of approximately 85%, with disagreements largely due to minor errors with essential agreement, is also acceptable under CLSI [57,58].

### 2.2. Heteroresistance

Among all 193 specimens and 3550 organism–antibiotic combinations tested, heteroresistance was detected by P-AST in 39 instances (Table 5 and Table 6. In these cases, P-AST would have correctly resulted with “resistant” while BMD Isolate AST would have resulted with “sensitive” due to its inability to detect heteroresistance. Contingency tables for analysis by microorganism subset can be found in Supplemental Tables S7–S8. P-AST Major Errors decreased after correcting for these instances as detailed in Table 7.

These heteroresistance cases were BMD Isolate AST VMEs, since BMD reported them as sensitive despite the presence of heteroresistance, with a VME rate of 1.1% overall, 1.2% for 2 organisms, and 0.8% for ≥3 organisms.

The relative contribution of each antibiotic class to the total percentage of each type of error (VME, ME, and mE) by P-AST, including heteroresistance-corrected MEs, is shown in Table 8. No one antibiotic or antibiotic class produced large numbers of all types of errors. The penicillins (including ampicillin, amoxicillin/clavulanate, ampicillin/sulbactam, and piperacillin/tazobactam) had the most of all types of errors, and were the largest source of heteroresistance.

### 2.3. Impact of Additional Organisms

We next sought to assess the association between the number of organisms present and the average number of “sensitive” antibiotics per specimen from a large database of real-world clinical test results. This database analysis included de-identified P-AST results for clinical urine specimens from 57,084 patients with presumptive UTI diagnoses and consistent ICD-10-CM codes. The specimens were provided mostly by older adults with a median age of 72.4 years and a mean age of 68.6 (SD = 17.0). Only specimens with a microbial density ≥ 10,000 cells/mL had their P-AST results analyzed. The data were first analyzed to assess the impact of increasing numbers of non-fastidious organisms. Increasing numbers of fastidious organisms were then assessed in the presence of one non-fastidious organism to determine whether they had any impact on average susceptibility results per sample.

**Figure 2:**
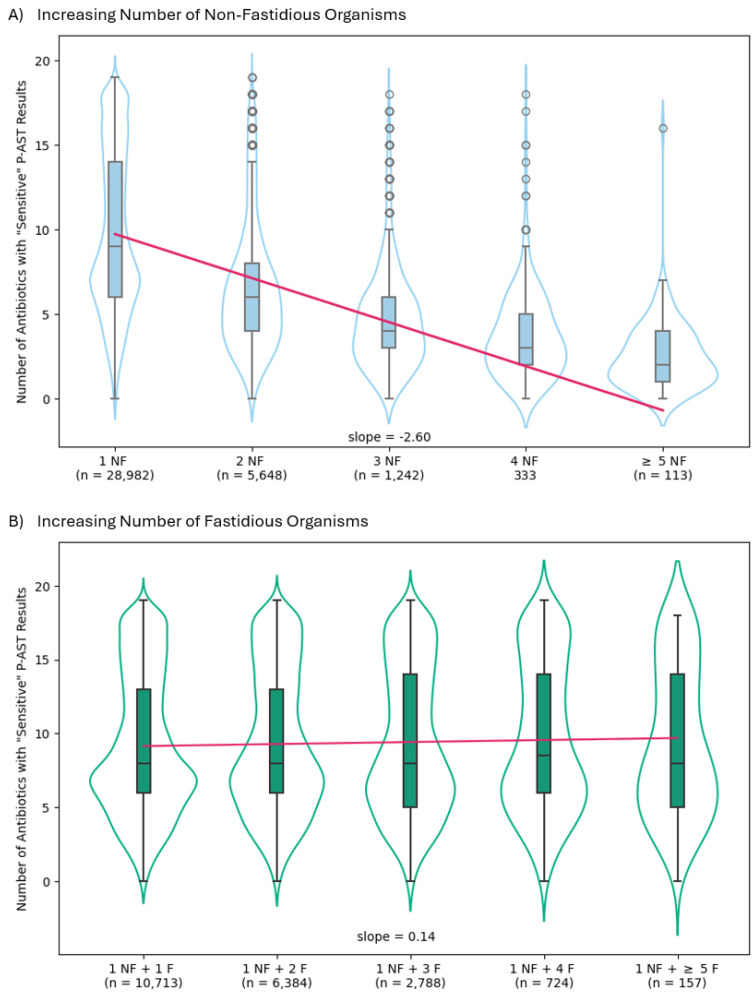
Effect of Non-Fastidious and Fastidious Organisms on Number of Sensitive Antibiotic Results in Polymicrobial Infections. Specimens are divided into groups based on the number and nature (NF = non-fastidious and F = fastidious) organisms detected. (**A**) The addition of non-fastidious organisms: 1 NF, 2 NF, 3 NF, 4 NF, and ≥5 NF (light blue) and (**B**) the addition of fastidious organisms: 1 NF + 1 F, 1 NF + 2 F, 1 NF + 3 F, 1 NF + 4 F, and 1 NF + ≥5 F (teal green). The *n* for each subgroup is included below the labels. The number of antibiotics, out of 19 possible, with “sensitive” AST results is plotted on the *y*-axis. Boxes extend from the first to third quartiles with a line at the median and whiskers extending to 1.5 IQR or maximum. Outliers are plotted as open circles. Kernel density estimation (KDE) violin plots for each data group are shown around each box plot. A fitted linear regression of all data points is displayed as a red line.

Each additional non-fastidious organism in the urine specimen was associated with a drop in the average number of sensitive antibiotic results (Figure 2A, Supplemental Table S9). The overall average dropped from 9.8 in the presence of one organism to 2.5 for five or more organisms. This is reflected in the moderate effect size of 0.1196 (*p* < 0.0001). In contrast, the addition of one or more fastidious organisms to specimens with one non-fastidious organism had a negligible impact on the number of sensitive P-AST results. The overall average of 9.8 sensitive antibiotics for one non-fastidious organism remained close, at 9.3, even when five or more fastidious organisms were present. The effect size was very low at 0.0004 (p = 0.0207) (Figure 2B, Supplemental Table S9).

## 3. Discussion

Novel diagnostic assays for complicated and recurrent UTIs are an important tool in closing the clinical gap caused by the known limitations of SUC. This is especially important in polymicrobial UTIs, where both the inability of SUC to grow multiple organisms and the discounting of multiple organisms found in a urine sample have resulted in high rates of missed organisms and mixed flora/contamination results. However, these novel assays need to demonstrate clinical validity and utility compared to the current standard of care. The P-AST method, which measures the pooled antibiotic susceptibility for presumptive UTI samples, has already been validated using CLSI criteria for monomicrobial specimens [56]. In this study, P-AST was validated for polymicrobial specimens, including an analysis that showed the impact of additional fastidious and non-fastidious organisms.

Using CLSI standard validation protocols in consecutive clinical samples, the P-AST assay met the criteria when compared to standard AST methods [57,58]. The essential agreement between P-AST and urine culture (UC) was >90%, while Very Major Errors and Major Errors were <3%, in polymicrobial samples with two or more non-fastidious organisms present. Minor errors in this study were within laboratory limits, and categorical agreement was approximately 85%. These are acceptable within CLSI standards, especially since most discrepancies were minor errors with essential agreement [57,58].

Comparable clinical validity studies are typically conducted when (1) a new resistance mechanism is identified as clinically relevant, (2) a new antibiotic becomes available, or (3) a new test system is being validated. Validations of commonly utilized commercially available AST tests, including automated systems like VITEK, Phoenix, and Microscan, are relevant comparators for this type of study. They have reported very major error rates > 3.0% [59,60], major error rates > 3.0% [59,60,61], and essential agreement as low as 71% [59], showing that even these FDA-approved products do not always meet CLSI criteria for all metrics. The results of this study on the P-AST assay, which do meet these criteria, are equivalent or superior in comparison. Other published studies comparing the essential/categorical agreements and errors of the disk diffusion method against broth microdilution frequently report significantly greater disagreement than observed between P-AST and BMD in this study [61,62,63,64,65,66,67]. Specifically, categorical agreement as low as 50% [68] and very major error rates as high as 53.3% [69] have been reported for these comparisons. Manual AST with gradient or E-test methods has been reported to produce even lower agreement with BMD than standard DD AST [59,70]. The P-AST data in this study are superior or equivalent to these studies as well.

When heteroresistance was accounted for, the P-AST assay performed better with overall MEs dropping to 1%. The experimentally verified heteroresistance rate in this study accounts for some of the discrepancies between methods in polymicrobial specimens. Significant prior literature details the importance of heteroresistance in determining the true susceptibility of infections and the limitations of isolate testing to detect it [52,53,54,55,71,72,73,74,75,76,77]. Multiple factors, including microbial interactions, likely account for the remaining discordance [5,78].

This study allowed for an apples-to-apples comparison of standard AST and P-AST by using specimens where culture was capable of identifying the same pathogens as M-PCR and the resistance of all identified organisms was accounted for (by categorizing antibiotics as resistant when any one of the pathogens is resistant, Figure S1). The high concordance between P-AST and standard isolate AST methods in these cases demonstrates the validity of using a resuspended pellet to measure the pooled antibiotic susceptibility with P-AST, and that it is the identified pathogens that are driving the P-AST results.

Importantly, in both this study and the study of monomicrobial samples [56], there was a high concordance between P-AST and standard AST methods when the identified pathogen(s) were the same between the two approaches. The low rates of Major Errors, which should be high if multiple contaminants or commensals were contributing additional resistance to the pooled susceptibility results, is especially indicative of this. Overall, the data demonstrate that the pooled antibiotic susceptibility method results are driven by pathogens identified and not by other potentially confounding organisms in the pellet, at least for UTI urine specimens. It is possible that mid-stream voided and catheterized UTI samples do not carry a significant burden of non-pathogenic contaminants or that such contaminants do not have an appreciable impact on antibiotic susceptibility.

Since P-AST measures the pooled susceptibility using a resuspended pellet from the urine specimen, it is important to assess the impact of an increasing number of pathogens in the pooled organism resuspension. It would be expected that additional identification of non-fastidious organisms would make resistance more likely, since the P-AST pooled result reports antibiotics as sensitive only if all non-fastidious organisms are susceptible. In contrast, fastidious organisms do not grow in either BMD or in P-AST and antibiotic susceptibility is not assessed for them. Therefore, it would not be expected that additional fastidious organisms would impact the results.

Using a large real-world database of de-identified clinical sample results (Figure 2), we observed that increasing numbers of identified non-fastidious species were indeed associated with a major reduction in the number of susceptible antibiotics. The mean number of susceptible antibiotics with one organism was 9.8, steadily declining down to 2.5 with five or more organisms. The addition of fastidious organisms, irrespective of the number, to a non-fastidious organism had no appreciable effect on the mean number of susceptible antibiotics. The mean number of susceptible antibiotics with one non-fastidious organism was 9.8, which was not meaningfully different from the mean of 9.3 when five or more fastidious organisms were added. Due to the large number of samples (*n* = 57,084), there was a significant *p*-value for both sets, but the effect size of adding fastidious organisms was very small (effect size = 0.0004, *p* = 0.0207) while it was moderate when additional non-fastidious organism (effect size = 0.1196, *p* < 0.0001).

These results show an association between polymicrobial infections and increasing multi-drug resistance, which has previously been demonstrated [32,36,40,41,43,44,46,47,79]. Since SUC has known limitations in detecting polymicrobial infections, it could be providing incorrect results of “sensitive” by failing to identify resistant pathogens in the sample. The ability of P-AST to account for increasing resistance with increasing numbers of identified organisms differentiates it from SUC.

A previous study has also demonstrated that P-AST can detect changes in antibiotic susceptibility resulting from interactions between two microorganisms [5]. These interaction effects have also been clearly demonstrated in vivo. For example, a mouse pyelonephritis model of coinfection by *P. aeruginosa* and *E. faecalis* had higher resistance to β-lactam antibiotics compared to *P. aeruginosa* monomicrobial infection [80].

The number of non-fastidious organisms per sample in the 193 consecutive samples tested had a very similar distribution as compared to the real-world data, meaning that these results are applicable to the general patient population tested by Guidance UTI. The majority of polymicrobial samples had two non-fastidious organisms, approximately 15% had three, and only approximately 5% had four or more.

This study was designed with a strong bias in favor of urine culture, both by excluding cases where culture did not grow the pathogen detected by M-PCR in the urine, and by providing results without correcting for heteroresistance. It also created a fair match between the two methods by using EQUC to grow organisms in cases where they were missed by SUC and then testing those isolates using standard AST methods (Figure S1). Using these tests in parallel on the same polymicrobial UTI urine specimens, the P-AST methodology passed the validation criteria set by CLSI.

These results demonstrate that the testing of the antibiotic susceptibility of a polymicrobial urine specimen pellet accurately reflects the combined antibiotic susceptibility profiles of each species isolated from polymicrobial urine specimens. P-AST offers distinct advantages when measuring antimicrobial susceptibility in polymicrobial specimens. The novel use of a microbial pellet and resazurin-based fluorescent growth indicator for susceptibility testing enables the rapid detection of heteroresistant cases, as demonstrated here. The combined M-PCR/P-AST assay also has prior evidence of improving patient outcomes compared to SUC, including reductions in hospitalizations, ER and Urgent care visits, and lower rates of recurrence [10].

P-AST, a component of the Guidance^®^ UTI assay, demonstrates high essential agreement and low rates of very major and major errors within the thresholds established by CLSI for AST testing in polymicrobial samples. Combined with prior evidence that it also reduced negative outcomes in complicated and recurrent UTI cases and passed CLSI validation metrics in monomicrobial samples, this test may be considered for the management of complicated and recurrent UTIs.

## 4. Materials and Methods

### 4.1. Study Design

This study is an analysis of consecutive fresh clinical urine specimens submitted from providers across the United States of America (USA) with sufficient volume (minimum 2 mL) in boric acid stabilizer for diagnostic testing along with ICD-10-CM codes consistent with a diagnosis of UTI. The analysis included all polymicrobial specimens with the same two or more non-fastidious microorganism species identified by both M-PCR and urine culture at a density ≥ 10,000 cells/mL or CFUs/mL.

Samples for the study were collected via a biobank in which remnant urine specimens remaining after routine clinical testing were de-identified and assigned a unique repository code label associated only with the subject’s age, sex, and any associated ICD-10-CM code(s). Therefore, the Western Institutional Review Board deemed the use of the data to be exempt under 45 CFR § 46.104(d)(4) as the information was used in a manner that the identity of the subject could not be readily ascertained directly or through identifiers linked to the subjects, the subject was not contacted, and the investigator did not re-identify subjects. 

### 4.2. Bacterial Identification with Multiplex-Polymerase Chain Reaction (M-PCR)

The M-PCR assay was performed as previously described [10]. Briefly, DNA extracted from urine samples using the King Fisher/MagMAX™ automated DNA extraction instrument and the MagMAX™ DNA Multi-Sample Ultra Kit (Thermo Fisher, Carlsbad, CA, USA) was mixed with a universal PCR master mix and amplified using TaqMan technology in a Life Technologies 12K Flex 112-format Open Array System (Thermo Fisher Scientific, Wilmington, NC, USA). Probes and primers were used to detect the following 23 bacterial species and three bacterial groups: *Acinetobacter baumannii* (*A. baumannii*); *Actinotignum schaalii* (*A. schaalii*); *Aerococcus urinae* (*A. urinae*); *Alloscardovia omnicolens* (*A. omnicolens*)*; Citrobacter freundii* (*C. freundii*); *Citrobacter koseri* (*C. koseri*); *Corynebacterium riegelii* (*C. riegelii*)*; Enterococcus faecalis* (*E. faecalis*); *Enterococcus faecium* (*E. faecium*)*; Escherichia coli* (*E. coli*); *Gardnerella vaginalis* (*G. vaginalis*); *Klebsiella oxytoca* (*K. oxytoca*); *Klebsiella pneumoniae* (*K. pneumoniae*); *Morganella morganii* (*M. morganii*); *Mycoplasma hominis* (*M. hominis*); *Pantoea agglomerans* (*P. agglomerans*); *Proteus mirabilis* (*P. mirabilis*); *Providencia stuartii* (*P. stuartii*); *Pseudomonas aeruginosa* (*P. aeruginosa*); *Serratia marcescens* (*S. marcescens*); *Staphylococcus aureus* (*S. aureus*); *Streptococcus agalactiae* (*S. agalactiae*); *Ureaplasma urealyticum* (*U. urealyticum*); Coagulase Negative Staphylococci (CoNS), which includes *Staphylococcus epidermidis*, *Staphylococcus haemolyticus, Staphylococcus lugdunenesis,* and *Staphylococcus saprophyticus* (*S. saprophyticus*); the Enterobacter Group, which includes *Klebsiella aerogenes* (*K. aerogenes*) (*formally known as Enterobacter aerogenes*) and *Enterobacter cloacae* (*E. cloacae*); and Viridans Group Streptococci (VGS), which includes *Streptococcus anginosus*, *Streptococcus oralis*, and *Streptococcus pasteuranus.* Negative extraction controls containing only the buffers used in the process are used to detect contamination during the DNA extraction, are performed with every sample batch, and are carried throughout the entire Guidance^®^ UTI assay process. The positive extraction control, *Bacillus atrophaeus* (a bacterial organism that is not part of the urobiome), is spiked into each sample and is carried through to M-PCR as a control against PCR inhibition.

### 4.3. Bacterial Identification with Standard Urine Culture (SUC) and Expanded Quantitative Urine Culture (EQUC)

Bacterial identification by SUC was performed as previously described [6]. Briefly, a 1 µL sterile plastic loop was used to inoculate both a blood agar plate (BAP) and a colistin and nalidixic acid agar/MacConkey agar (CNA/MAC) plate with one loop of urine specimen on each side of the CNA/MAC plate. Plates were all incubated at 35 °C in a non-CO_2_ incubator for 18 h and examined for growth.

If standard SUC results were non-specific, such as “contaminated” or “mixed flora”, or when SUC and M-PCR organism identifications did not match, the specimen was re-plated under EQUC conditions to enable identification of all individual species present. EQUC uses Blood Agar (BAP), Chocolate Agar, Colistin Nalidixic Acid Agar (CNA), and Anaerobic BAP plates cultured at 35 °C. EQUC conditions also require plating a larger volume of the specimen (50 µL for EQUC compared to a 1 µL loop for SUC) to facilitate detection of organisms present at lower concentrations. Organisms cultured by EQUC conditions were then isolated and their identity was confirmed via M-PCR.

### 4.4. Pooled Antibiotic Susceptibility Testing (P-AST)

The resazurin-fluorescence-based P-AST test component was performed as described previously [16]. Briefly, 1 mL of urine specimen was transferred into a microcentrifuge tube. After centrifugation, the urine supernatant was discarded, and the microbial pellet was suspended with 1 mL of Mueller Hinton Growth (MHG) media and then incubated for 6 h at 35 °C in a non-CO_2_ incubator. At the end of the incubation, samples reaching a predetermined density threshold were diluted with MHG media in a 50 mL conical tube to a final concentration of approximately 500 thousand cells/mL. The diluted sample was inoculated into a 96-well plate pre-loaded with multiple concentrations of 19 antibiotics and incubated at 35 °C for 12–16 h, along with the control plates. Resazurin was used as a fluorescent probe, and the fluorescent density of the samples was measured on an Infinite M Nano+ Microplate Reader (TECAN, Männedorf, Switzerland) to measure cell growth at the end of the incubation.

The P-AST assay includes a blank plate with wells containing only MH media as a negative control for the fluorescent growth indicator, resazurin. Three organisms with known antibiotic resistance profiles (*E. coli*, *P. stuartii*, and *A. baumannii*) serve as positive controls.

P-AST applies the most restrictive Minimum Inhibitory Concentration (MIC) breakpoint among all detected non-fastidious organisms. These breakpoints are from the most recent CLSI guidelines M100 34th edition [81] and are updated at least yearly. This approach ensures that the susceptibility classification reflects the lowest susceptibility threshold among the detected organisms within the sample (see example scenario in Supplemental Table S4).

### 4.5. Broth Microdilution (BMD) Antibiotic Susceptibility Testing

BMD AST was performed on isolates from the SUC and/or EQUC plates following standard procedures outlined in the CLSI M07 12th edition (2024) [82]. Briefly, individual isolates were suspended in MHG media and adjusted to 0.5 McFarland. This preparation was diluted to a final density of 5 × 10^5^ CFUs/mL in MHG that was supplemented with relevant antibiotic dilutions in a 96-well microtiter plate. These plates were incubated at 35 °C in a non-CO_2_ incubator for 16 h, after which the turbidity of each well was measured against positive and negative growth controls.

### 4.6. Disk Diffusion (DD) Antibiotic Susceptibility Testing

DD AST was performed initially on organisms isolated by SUC and/or EQUC, but during the heteroresistance analysis, DD AST was performed on diluted subculture from a P-AST well displaying resistance. Briefly, the suspended isolate or pre-cultured well dilution was grown as a lawn on an MH plate with a single antibiotic disk, and clearance zones were measured and interpreted after incubation at 35 °C for 16 h, according to the CLSI M100 34th edition [81].

### 4.7. Four-Times Antibiotic Concentration Culture for Resistance Verification

Briefly, the contents of each P-AST-resistant antibiotic were diluted with fresh Mueller–Hinton (MH) media at a 1:10 ratio for preculture at 35 °C overnight. The preculture was diluted with saline, adjusted to 0.5 McFarland, and plated using a 10 µL loop on MH impregnated with antibiotic at a concentration of 4× the highest Minimum Inhibitory Concentration (MIC) tested in the resistant P-AST well. Any growth after 12–18 h incubation at 35 °C was interpreted as confirming the resistant phenotype.

### 4.8. Generating Overall Isolate AST Profiles for Organisms Isolated by SUC and/or EQUC

To ensure that this study provided equivalent comparisons between the isolate AST and P-AST methods, any organisms identified by M-PCR and not by SUC were isolated by EQUC and had BMD run to determine the standard antibiotic sensitivity profile. The antibiotic profiles were then aligned, and the overall AST result was determined to generate an appropriate comparator. To generate the overall isolate AST profile, the following rules were applied: (1) If all of the organisms identified were determined to be sensitive to an antibiotic, the overall result was designated to be “sensitive”; (2) if any one organism was determined to be resistant to an antibiotic using the methods outlined above, the overall result was determined to be “resistant”. This generated an overall antibiotic susceptibility profile that was then compared with P-AST (Supplemental Figure S1).

### 4.9. Analysis Workflow for Resolving Discrepant Results and Detecting Heteroresistant Phenotypes

Similarly to the workflow previously described in the monomicrobial comparisons [56], urine samples were subjected, in parallel, to standard urine culture and M-PCR for microbial identification and quantification. Specimens negative for microbial detection by SUC and/or M-PCR were excluded. Monomicrobial specimens were also excluded from this analysis. If standard SUC results were non-specific, such as “contaminated” or “mixed flora”, or when SUC and M-PCR organism identifications did not match, the specimen was re-plated for EQUC to enable identification of all individual species present (Figure 2).

For specimens with the same organisms identified by M-PCR and by urine culture (SUC and/or EQUC, hereafter referred to simply as UC), antibiotic susceptibility testing was then conducted. Isolates from UC were tested by BMD. If a resistant result was obtained for any species isolated by UC for each specimen, the overall BMD AST result was deemed resistant. Simultaneously, P-AST was conducted using the urine specimen pellet. Susceptibility results were then compared between P-AST and the combined BMD AST result. Cases in which susceptibility results were discrepant between the two techniques underwent DD AST using the isolates from UC to resolve the discrepancy (Figure 2). When BMD and DD results were in agreement, the AST result was defined as “Isolate AST Consensus”. If BMD and DD results were conflicting, the sample was excluded. No further analysis was performed for specimens with P-AST-sensitive and BMD/DD consensus-resistant discrepant results.

Specimens with P-AST-resistant and BMD/DD consensus-sensitive results underwent further testing to identify heteroresistant phenotypes. Briefly, a specimen from the resistant P-AST culture well was simultaneously plated onto BAP, onto MH with 4X antibiotic concentration, and grown into a lawn on MH for DD AST. If no growth was observed on these three plates, the “Isolate AST Consensus” was affirmed as sensitive, and the P-AST results were deemed falsely resistant. If growth was observed in ≥2/3 test plates, the resistant P-AST result was affirmed, and the BMD/DD initial “Isolate AST Consensus” result determination was deemed falsely sensitive, representing a case of heteroresistance detected by the novel P-AST technique and missed by the standard isolate AST techniques BAP and DD. The “Heteroresistance-Corrected Consensus” was then updated to reflect the heteroresistant cases.

### 4.10. Analysis of Effects of Additional Organisms

For this analysis, we utilized a database of de-identified P-AST results for clinical urine specimens. We merged the data from our lab server and our laboratory information system (LIS) based on de-identified urine sample barcodes. The demographic data (age and sex of the patient only, as well as associated ICD-10-CM codes) were collected from the LIS, and the organism(s) identification and microbial density, resistance gene detection, and phenotypic antibiotic susceptibility data were all collected from the lab server. We filtered out all orphan records that only exist in one of the two systems, any non-clinical test data or alternative study data, and any samples missing date of birth information or a signed, dated, and complete result report. We included data only for cases submitted under a presumptive diagnosis of UTI with sample accession dates between 27 June 2022 and 23 November 2024, bringing the total number of cases to 76,257. After selection for polymicrobial specimens (exclusion of negative results and monomicrobial specimens), the final *n* for the analysis was 57,084.

### 4.11. Statistical Analyses

Metrics of P-AST validation were calculated according to CLSI standards [57,58]. Essential agreement (EA%) = Number of tests with minimum inhibitory concentration (MIC) within ± one two-fold dilution/total tests × 100. Categorical agreement (CA%) = Number of tests with same category results/total tests × 100. Very major errors (VME%) = Number of tests where the P-AST result is “S”, and the “Consensus” result is “R”/total tests × 100. Major errors (ME%) = Number of tests where the P-AST result is “R”, and the “Consensus” result is “S”/total tests × 100. Minor errors (mE%) = % of tests where (1) the P-AST result is “I” and the “Consensus” result is either “S” or “R” OR (2) the P-AST result is either “S” or “R” and the “Consensus” result is “I”. In the heteroresistance-corrected analysis, cases of heteroresistance were removed from the count of MEs by P-AST and added to the count of VMEs for BMD and DD AST. For all measures, 95% confidence intervals (95% CI) were calculated using the Agresti–Coull method. Because the heteroresistance-corrected consensus incorporates DD AST results, comparisons of essential agreement (including EA% and mEs with essential agreement), which require MICs, were not possible. Metrics aside from VMEs (which are due to heteroresistance correction) are not applicable for Isolate BMD AST, since it is the reference standard comparator for this study.

Differences in the number of antibiotics with “sensitive” AST results across groups with additional non-fastidious organisms and across groups with additional fastidious organisms were compared using the Kruskal–Wallis test. The effect size of the test was reported as eta-squared using the formula eta-squared = (H − *k* + 1)/(*n* − *k*) where H is the Kruskal–Wallis test statistic, *k* is the number of groups, and *n* is the total number of data points. Simple linear regression was used to examine the linear relationship between the number of non-fastidious or fastidious organisms and the number of sensitive antibiotics. The independent variable was the number of non-fastidious or fastidious organisms. The dependent variable was the number of sensitive antibiotics. An intercept was added when fitting the regression line.

The subset analyses of Gram-positive and Gram-negative cases were performed on specimens where all non-fastidious organisms had the same Gram staining. The Gram staining of fastidious organisms was not considered, and cases with mixed Gram-positive and Gram-negative non-fastidious organisms were excluded from the subset analyses.

## 5. Conclusions

P-AST, a unique component of the Guidance^®^ UTI assay which rapidly measures the susceptibility of a bacterial pellet of cultivable organisms from a urine specimen, demonstrates high Essential Agreement (>90%) and low Very Major and Major error rates (<3%) within the thresholds established by CLSI for AST testing in polymicrobial UTIs. Minor Errors and Categorical Agreement also meet criteria, especially since most discrepancies are minor errors with essential agreement. P-AST demonstrates the capacity to detect heteroresistance phenotypes which are not detected by isolate AST methods. P-AST also demonstrates the association of increasing numbers of non-fastidious organisms with increasing multidrug resistance and no appreciable change in antibiotic sensitivity in the presence of fastidious organisms.

## Figures and Tables

**Table 1:** Distribution of Non-fastidious and Total Number of Organisms for All Polymicrobial Specimens.

Number of Organisms	Specimen Count *n* (% of all 193 Specimens)
Number of Non-fastidious Organisms Only	
2	153 (79%)
3	29 (15%)
4	7 (4%)
≥5	4 (2%)
Total Number of Organisms (Fastidious and Non-fastidious)	
2	88 (46%)
3	44 (23%)
4	39 (20%)
≥5	22 (11%)

**Table 2:** ICD-10-CM Codes Associated with Study Specimens.

ICD-10-CM Code	Description	*n* (%)
**(A)** All Cases with Two or More Non-fastidious Organisms (*n* = 193)		
N39.0	Urinary tract infection, site not specified	148 (76.7%)
R30.0	Dysuria	23 (11.9%)
Z87.440	Personal history of urinary (tract) infections	9 (4.7%)
R82.998	Other abnormal findings in urine	7 (3.6%)
R31.0	Gross hematuria	6 (3.1%)
Others	NA	43 (22.3%)
**(B)** All Cases with Exactly Two Non-fastidious Organisms (*n* = 153)		
N39.0	Urinary tract infection, site not specified	117 (76.5%)
R30.0	Dysuria	21 (13.7%)
Z87.440	Personal history of urinary (tract) infections	6 (3.9%)
R82.998	Other abnormal findings in urine	5 (3.3%)
R31.0	Gross hematuria	4 (2.6%)
Others	NA	27 (17.6%)
**(C)** All Cases with Three or More Non-fastidious Organisms (*n* = 40)		
N39.0	Urinary tract infection, site not specified	31 (77.5%)
Z87.440	Personal history of urinary (tract) infections	3 (7.5%)
N40.1	Benign prostatic hyperplasia with lower urinary tract symptoms	3 (7.5%)
R30.0	Dysuria	2 (5.0%)
R31.0	Gross hematuria	2 (5.0%)
Others	NA	15 (37.5%)

**Table 3:** P-AST Performance Contingency Table for All Cases with Two or More Non-fastidious Organisms.

	P-AST Sensitive	P-AST Intermediate	P-AST Resistant	Total
Isolate AST Consensus Sensitive	680 (19.2%)	85 (2.4%)	74 (2.1%)	839 (23.6%)
Isolate AST Consensus Intermediate	127 (3.6%)	92 (2.6%)	109 (3.1%)	328 (8.3%)
Isolate AST Consensus Resistant	39 (1.1%)	94 (2.6%)	2250 (63.4%)	2383 (67.1%)
Total	846 (23.8%)	271 (7.6%)	2433 (68.5%)	3550 (100.0%)

P-AST = pooled antibiotic susceptibility testing. AST = antibiotic susceptibility testing.

**Table 4:** P-AST Versus Isolate AST Performance Across All Cases Two or More Non-fastidious Organisms.

	All Specimens (*n* = 193 Specimens)		All Gram-Negative (*n* = 33 Specimens)		All Gram-Positive (*n* = 15 Specimens)		2 Non-Fastidious (*n* = 153 Specimens)		≥3 Non-Fastidious (*n* = 40 Specimens)	
	*n*	% (95% CI)	*n*	% (95% CI)	*n*	% (95% CI)	*n*	% (95% CI)	*n*	% (95% CI)
Essential Agreement (EA%)	3334	93.9% (93.1%, 94.7%)	578	93.8% (91.6%, 95.5%)	257	93.5% (89.8%, 95.9%)	2641	94.0% (93.0%, 94.8%)	693	93.6% (91.6%, 95.2%)
Very Major Errors (VMEs)	39	1.1% (0.8%, 1.5%)	7	1.1% (0.5%, 2.4%)	3	1.1% (0.2%, 3.3%)	28	1.0% (0.7%, 1.4%)	11	1.5% (0.8%, 2.7%)
Major Errors (MEs)	74	2.1% (1.7%, 2.6%)	17	2.8% (1.7%, 4.4%)	5	1.8% (0.7%, 4.3%)	56	2.0% (1.5%, 2.6%)	18	2.4% (1.5%, 3.8%)
Minor Errors (mEs)	415	11.7% (10.7%, 12.8%)	69	11.2% (8.9%, 13.9%)	29	10.5% (7.4%, 14.8%)	319	11.4% (10.2%, 12.6%)	96	13.0% (10.7%, 15.6%)
Minor Errors w/Essential Agreement	314	8.8% (8.0%, 9.8%)	55	8.9% (6.9%, 11.5%)	19	6.9% (4.4%, 10.6%)	235	8.4% (7.4%, 9.4%)	79	10.7% (8.6%, 13.1%)
Categorical Agreement (CA%)	3022	85.1% (83.9%, 86.3%)	523	84.9% (81.9%, 87.5%)	238	86.5% (82.0%, 90.1%)	2407	85.7% (84.3%, 86.9%)	615	83.1% (80.2%, 85.6%)

P-AST = pooled antibiotic susceptibility testing. AST = antibiotic susceptibility testing. EA% describes the agreement between MIC values. VMEs are also known as false-susceptibility errors. MEs are also known as false-resistance errors. mEs are discrepancies between categorical calls involving an intermediate “I” call by either method. CA% describes the agreement between the susceptible, intermediate, and resistant calls. Details of these measures and their calculations are in the statistical analysis section of Methods.

**Table 5:** P-AST Performance Contingency Table for All Cases with Two or More Non-fastidious Organisms.

	P-AST Sensitive	P-AST Intermediate	P-AST Resistant	Total
Heteroresistance-Corrected Consensus Sensitive	680 (19.2%)	85 (2.4%)	35 (1.0%)	800 (22.5%)
Heteroresistance-Corrected Consensus Intermediate	127 (3.6%)	92 (2.6%)	109 (3.0%)	328 (8.3%)
Heteroresistance-Corrected Consensus Resistant	39 (1.1%)	94 (2.6%)	2289 (64.5%)	2422 (68.2%)
Total	846 (23.8%)	271 (7.6%)	2433 (68.5%)	3550 (100.0%)

P-AST = pooled antibiotic susceptibility testing. AST = antibiotic susceptibility testing.

**Table 6:** Isolate BMD AST Performance Contingency Table for All Cases with Two or More Non-fastidious Organisms.

	BMD AST Sensitive	BMD AST Intermediate	BMD AST Resistant	Total
Heteroresistance-Corrected Consensus Sensitive	800 (22.5%)	0 (0.0%)	0 (0.0%)	800 (22.5%)
Heteroresistance-Corrected Consensus Intermediate	0 (0.0%)	328 (9.2%)	0 (0.0%)	328 (9.2%)
Heteroresistance-Corrected Consensus Resistant	39 (1.1%)	0 (0.0%)	2383 (67.1%)	2422 (68.2%)
Total	839 (23.6%)	328 (9.2%)	2383 (67.1%)	3550 (100.0%)

BMD AST = broth microdilution antibiotic susceptibility testing.

**Table 7:** P-AST Performance After Correction for Heteroresistance Analysis.

	All Specimens (*n* = 193 Specimens)				2 Non-Fastidious (*n* = 153 Specimens)				≥3 Non-Fastidious (*n* = 40 Specimens)			
	Before Correction		Heteroresistance- Corrected		Before Correction		Heteroresistance- Corrected		Before Correction		Heteroresistance- Corrected	
	*n*	% (95% CI)	*n*	% (95% CI)	*n*	% (95% CI)	*n*	% (95% CI)	*n*	% (95% CI)	*n*	% (95% CI)
Major Errors (MEs)	74	2.1% (1.7%, 2.6%)	35	1.0% (0.7%, 1.4%)	56	2.0% (1.5%, 2.6%)	23	0.8% (0.5%, 1.2%)	18	2.4% (1.5%, 3.8%)	12	1.6% (0.9%, 2.8%)

P-AST = pooled antibiotic susceptibility testing. MEs are also known as false-resistance errors. Details of ME calculations are in the statistical analysis section of the Methods.

**Table 8:** P-AST Errors and Heteroresistance by Antibiotic Class.

Antibiotic Class	VME	ME	Heteroresistance- Corrected ME	mE
Penicillins [ampicillin, amoxicillin/clavulanate, ampicillin/sulbactam, and piperacillin/tazobactam]	0.4%	1.0%	0.5%	3.0%
Cephalosporins [cefaclor, cefazolin, cefepime, cefoxitin, ceftazidime, and ceftriaxone]	0.2%	0.2%	0.0%	0.8%
Fluoroquinolones [ciprofloxacin and levofloxacin]	0.1%	0.1%	0.0%	2.5%
Phosphonic [fosfomycin]	0.2%	0.2%	0.1%	1.8%
Aminoglycoside [gentamicin]	0.1%	0.1%	0.0%	2.0%
Nitrofuran [nitrofurantoin]	0.1%	0.1%	0.2%	1.2%
Carbapenem [meropenem]	0.1%	0.1%	0.1%	0.1%
Tetracycline	0.1%	0.1%	0%	0.2%
Sulfonamide/ Dihydrofolate Reductase Inhibitor [sulfamethoxazole/trimethoprim]	0.0%	0.1%	0.0%	0.0%
Glycopeptide [vancomycin]	0.0%	0.1%	0%	0.1%
Total *	1.1%	2.1%	1.0%	11.7%

VME = very major error; ME = major error; mE = minor error. * Due to rounding, percentages may exceed the totals, which are the rate of each error type for all polymicrobial specimens (see Table 4 for VME, ME, and mE and Table 7 for Heteroresistance-Corrected ME).

## Data Availability

The original data presented in the study are openly available in FigShare at DOI: 10.6084/m9.figshare.27871479.

## Supplementary Materials

The following supporting information can be downloaded at: https://www.mdpi.com/article/10.3390/antibiotics14020143/s1; Supplemental Table S1: Example of Pooled Antibiotic Susceptibility Testing (P-AST) MIC Breakpoints for Ciprofloxacin and Gentamicin when *E. coli*, *P. aeruginosa*, and *S. aureus* are Detected; Supplemental Figure S1: Illustration of Overall Susceptibility Profile Determination With Multiple Isolates From Urine Culture; Supplemental Table S2: Relative Frequency of Bacterial Species and Groups; Supplemental Table S3: P-AST Performance Contingency Table for All Cases With Two or More Gram-Negative Non-fastidious Organisms; Supplemental Table S4: P-AST Performance Contingency Table for All Cases With Two or More Gram-Positive Non-fastidious Organisms; Supplemental Table S5: P-AST Performance Contingency Table for All Cases with Exactly Two Non-fastidious Organisms; Supplemental Table S6: P-AST Performance Contingency Table for All Cases with Three or More Non-fastidious Organisms; Supplemental Table S7A: Heteroresistance-Corrected P-AST Performance Contingency Table for All Cases with Exactly Two Non-fastidious Organisms; Supplemental Table S7B: Heteroresistance-corrected Isolate BMD AST Performance Contingency Table for All Cases with Exactly Two Non-fastidious Organisms; Supplemental Table S8A: Heteroresistance-corrected P-AST Performance Contingency Table for All Cases with Three or More Non-fastidious Organisms; Supplemental; Supplemental Table S8B: Heteroresistance-corrected Isolate BMD AST Performance Contingency Table for All Cases with Three or More Non-fastidious Organisms; Supplemental Table S9: Descriptive Statistics for Real-World Analysis of the Effect of Non-Fastidious and Fastidious Organisms on Number of Sensitive Antibiotic Results in Polymicrobial Infections.
