# Supplementary material for: Pooled Antibiotic Susceptibility Testing for Polymicrobial UTI Performs Within CLSI Validation Standards
Source: Antibiotics (Basel). 2025 Feb 1;14(2):143. doi: 10.3390/antibiotics14020143 (PMC11852178; doi:10.3390/antibiotics14020143)
Supplement: Supplementary file 1 [file antibiotics-14-00143-s001.zip › antibiotics-3390593-supplementary.pdf]

Supplemental Table S1. Example of Pooled Antibiotic Susceptibility Testing (P-AST) MIC Breakpoints for Ciprofloxacin and Gentamicin when *E. coli*, *P. aeruginosa*, and *S. aureus* are detected.

Organism	*E. coli*			*P. aeruginosa*			*S. aureus*			P-AST MIC Used		
	S	I	R	S	I	R	S	I	R	S	I	R
Ciprofloxacin	< 0.25	0.5	> 1	< 0.5	1	> 2	< 1	2	> 4	< 0.25	0.5	> 1
Gentamycin	< 2	4	> 8	< 4	8	> 16	< 4	8	> 16	< 2	4	> 8

Note: Minimum Inhibitory Concentration (MIC) values are in mg/mL. S = Susceptible, I = Intermediate, R = Resistant. The P-AST MIC is determined based on the lowest susceptibility breakpoint among the detected organisms.

For example, when *Escherichia coli*, *Pseudomonas aeruginosa*, and *Staphylococcus aureus* were detected in the pooled sample, the susceptibility determination was based on the lowest MIC breakpoint for resistance. If the P-AST MIC for ciprofloxacin was ≥ 1 mg/mL, the sample was classified as resistant, reflecting the CLSI breakpoint for *E. coli* resistance at this level, despite the breakpoints for *S. aureus* (≥ 4 mg/mL) and *P. aeruginosa* (≥ 2 mg/mL) being higher. Similarly, if the P-AST MIC for gentamycin was ≥ 8 mg/mL, the result was classified as resistant due to the resistance breakpoint for *E. coli* at this concentration, even though *P. aeruginosa* and *S. aureus* remained susceptible at this level.

Supplemental Figure S1. Illustration of Overall Susceptibility Profile Determination With Multiple Isolates From Urine Culture.

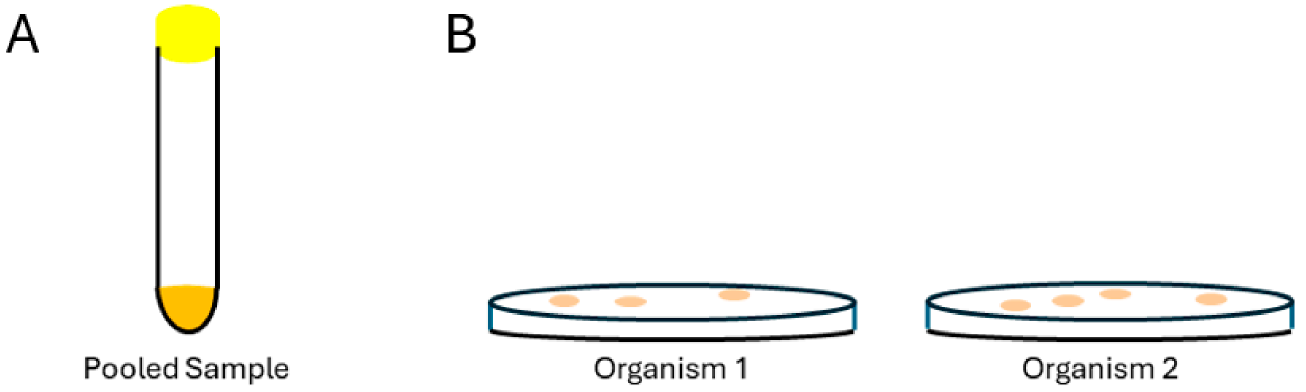

Antibiotic	P-AST Result
Ampicillin	Sensitive
Ciprofloxacin	Resistant
Gentamycin	Resistant

Antibiotic	Organism 1	Organism 2	Overall
Ampicillin	Sensitive	Sensitive	Sensitive
Ciprofloxacin	Resistant	Sensitive	Resistant
Gentamycin	Sensitive	Resistant	Resistant

Supplemental Figure S1. A) P-AST - Pooled susceptibility was determined by testing the pellet derived from a patient specimen. Organisms were not isolated from one another for this analysis. B) Isolate AST - To generate an overall comparison by isolate AST, all culture-derived organism isolates from each specimen (derived from SUC or from EQUC if not identified by SUC), individually had BMD AST performed to determine the antibiotic susceptibility profile for each organism (shown is an example with two organisms). In this example, the overall BMD AST result is concordant with the P-AST result (a matching antibiotic susceptibility profile) because, when each organism has contributed its specific resistance phenotype to the overall BMD AST result, the profile is identical to the P-AST result (tables).

Supplemental Table S2. Relative Frequency of Bacterial Species and Groups

Bacterial Species or Group	Frequency in Study Analysis		Frequency in Real World Clinical Database		*p*-value
	n	**%**	***n***	**%**	
**Non-Fastidious Organisms**					
Acinetobacter baumannii	2	1.0%	302	0.9%	0.705
Citrobacter freundii	9	4.7%	1664	5.2%	0.871
Citrobacter koseri	2	1.0%	789	2.5%	0.343
**Coagulase Negative Staphylococci**	23	11.9%	6442	20.1%	0.004
**Enterobacter Group**	18	9.3%	2999	9.4%	1.000
Enterococcus faecalis	152	78.8%	24273	75.9%	0.399
Enterococcus faecium*	9	4.7%	1434	4.5%	0.861
Escherichia coli	117	60.6%	19339	60.5%	1.000
Klebsiella oxytoca	8	4.1%	1924	6.0%	0.360
Klebsiella pneumoniae	50	25.9%	8797	27.5%	0.686
Morganella morganii	17	8.8%	2829	8.8%	1.000
Pantoea agglomerans*	0	0.0%	66	0.2%	1.0
Proteus mirabilis	20	10.4%	3841	12.0%	0.578
Providencia stuartii	2	1.0%	251	0.8%	0.666
Pseudomonas aeruginosa	14	7.3%	3659	11.4%	0.069
Serratia marcescens	4	2.1%	552	1.7%	0.579
Staphylococcus aureus	8	4.1%	1362	4.3%	1.000
Streptococcus agalactiae*	7	3.6%	1682	5.3%	0.416
**Viridans Group Streptococci***	30	15.5%	8274	25.9%	<0.001
**Fastidious Organisms***					
Actinotignum schaalii*	42	21.8%	8965	28.0%	0.054
Aerococcus urinae*	35	18.1%	8029	25.1%	0.025
Alloscardovia omnicolens*	11	5.7%	1761	5.5%	0.874
Corynebacterium riegelii*	3	1.6%	695	2.2%	0.803
Gardnerella vaginalis*	6	3.1%	3170	9.9%	<0.001
Mycoplasma hominis*	0	0.0%	210	0.7%	0.641
Ureaplasma urealyticum*	0	0.0%	636	2.0%	0.036

*P-AST component of the Guidance UTI assay is not performed on these organisms

Supplemental Table S3. P-AST Performance Contingency Table for All Cases With Two or More Gram-Negative Non-fastidious Organisms

	**P-AST Sensitive**	**P-AST Intermediate**	**P-AST Resistant**	*Total*
**Isolate AST Consensus Sensitive**	211 (34.3%)	15 (2.4%)	17 (2.8%)	243 (39.4%)
**Isolate AST Consensus Intermediate**	31 (5.0%)	17 (2.8%)	10 (1.6%)	58 (6.5%)
**Isolate AST Consensus Resistant**	7 (1.1%)	13 (2.1%)	295 (47.9%)	315 (51.1%)
Total	249 (40.4%)	45 (7.3%)	322 (52.3%)	616 (100.0%)

P-AST = pooled antibiotic susceptibility testing. AST = antibiotic susceptibility testing.

Supplemental Table S4. P-AST Performance Contingency Table for All Cases With Two or More Gram-Positive Non-fastidious Organisms

	**P-AST Sensitive**	**P-AST Intermediate**	**P-AST Resistant**	*Total*
**Isolate AST Consensus Sensitive**	72 (26.2%)	6 (2.2%)	5 (1.8%)	83 (30.2%)
**Isolate AST Consensus Intermediate**	6 (2.2%)	6 (2.2%)	9 (3.3%)	21 (8.4%)
**Isolate AST Consensus Resistant**	3 (1.1%)	8 (2.9%)	160 (58.2%)	171 (62.2%)
Total	81 (29.5%)	20 (7.3%)	174 (63.3%)	275 (100.0%)

P-AST = pooled antibiotic susceptibility testing. AST = antibiotic susceptibility testing.

Supplemental Table S5. P-AST Performance Contingency Table for All Cases with Exactly Two Non-fastidious Organisms

	P-AST Sensitive	P-AST Intermediate	P-AST Resistant	Total
Isolate AST Consensus Sensitive	603 (21.5%)	61 (2.2%)	56 (2.0%)	720 (25.6%)
Isolate AST Consensus Intermediate	96 (3.4%)	78 (2.8%)	85 (3.0%)	259 (8.5%)
Isolate AST Consensus Resistant	28 (1.0%)	77 (2.7%)	1,726 (61.4%)	1,831 (65.2%)
Total	727 (25.9%)	216 (7.7%)	1,867 (66.4%)	2,810 (100.0%)

P-AST = pooled antibiotic susceptibility testing. AST = antibiotic susceptibility testing.

Supplemental Table S6. P-AST Performance Contingency Table for All Cases with Three or More Non-fastidious Organisms

	P-AST Sensitive	P-AST Intermediate	P-AST Resistant	Total
Isolate AST Consensus Sensitive	77 (10.4%)	24 (3.2%)	18 (2.4%)	119 (16.1%)
Isolate AST Consensus Intermediate	31 (4.2%)	14 (1.9%)	24 (3.2%)	69 (7.4%)
Isolate AST Consensus Resistant	11 (1.5%)	17 (2.3%)	524 (70.8%)	552 (74.6%)
Total	119 (16.1%)	55 (7.4%)	566 (76.5%)	740 (100.0%)

P-AST = pooled antibiotic susceptibility testing. AST = antibiotic susceptibility testing.

Supplemental Table S7A. Heteroresistance-Corrected P-AST Performance Contingency Table for All Cases with Exactly Two Non-fastidious Organisms

	P-AST Sensitive	P-AST Intermediate	P-AST Resistant	Total
Heteroresistance-Corrected Consensus Sensitive	603 (21.5%)	61 (2.2%)	23 (0.8%)	687 (24.4%)
Heteroresistance-Corrected Consensus Intermediate	96 (3.4%)	78 (2.8%)	85 (3.0%)	259 (8.5%)
Heteroresistance-Corrected Consensus Resistant	28 (1.0%)	77 (2.7%)	1,759 (62.6%)	1,864 (66.3%)
Total	727 (25.9%)	216 (7.7%)	1,867 (66.4%)	2,810 (100.0%)

P-AST = pooled antibiotic susceptibility testing. AST = antibiotic susceptibility testing.

Supplemental Table S7B. Heteroresistance-corrected Isolate BMD AST Performance Contingency Table for All Cases with Exactly Two Non-fastidious Organisms

	BMD AST Sensitive	BMD AST Intermediate	BMD AST Resistant	Total
Heteroresistance-Corrected Consensus Sensitive	687 (24.4%)	0 (0.0%)	0 (0.0%)	687 (24.4%)
Heteroresistance-Corrected Consensus Intermediate	0 (0.0%)	259 (9.2%)	0 (0.0%)	259 (9.2%)
Heteroresistance-Corrected Consensus Resistant	33 (1.2%)	0 (0.0%)	1,831 (65.2%)	1,864 (66.3%)
Total	720 (25.6%)	259 (9.2%)	1,831 (65.2%)	2,810 (100.0%)

BMD AST = broth microdilution antibiotic susceptibility testing.

Supplemental Table S8A. Heteroresistance-corrected P-AST Performance Contingency Table for All Cases with Three or More Non-fastidious Organisms

	P-AST Sensitive	P-AST Intermediate	P-AST Resistant	Total
Heteroresistance-Corrected Consensus Sensitive	77 (10.4%)	24 (3.2%)	12 (1.6%)	113 (15.3%)
Heteroresistance-Corrected Consensus Intermediate	31 (4.2%)	14 (1.9%)	24 (3.2%)	69 (7.4%)
Heteroresistance-Corrected Consensus Resistant	11 (1.5%)	17 (2.3%)	530 (71.6%)	558 (75.4%)
Total	119 (16.1%)	55 (7.4%)	566 (76.5%)	740 (100.0%)

P-AST = pooled antibiotic susceptibility testing. AST = antibiotic susceptibility testing.

Supplemental Table S8B. Heteroresistance-corrected Isolate BMD AST Performance Contingency Table for All Cases with Three or More Non-fastidious Organisms

	BMD AST Sensitive	BMD AST Intermediate	BMD AST Resistant	Total
Heteroresistance-Corrected Consensus Sensitive	113 (15.3%)	0 (0.0%)	0 (0.0%)	113 (15.3%)
Heteroresistance-Corrected Consensus Intermediate	0 (0.0%)	69 (9.3%)	0 (0.0%)	69 (9.3%)
Heteroresistance-Corrected Consensus Resistant	6 (0.8%)	0 (0.0%)	552 (74.6%)	558 (75.4%)
Total	119 (16.1%)	69 (9.3%)	552 (74.6%)	740 (100.0%)

BMD AST = broth microdilution antibiotic susceptibility testing.

Supplemental Table S9. Descriptive Statistics for Real-World Analysis of the Effect of Non-Fastidious and Fastidious Organisms on Number of Sensitive Antibiotic Results in Polymicrobial Infections

	1 NF	2 NF	3 NF	4 NF	≥ 5 NF	1 NF + 1 F	1 NF + 2 F	1 NF + 3 F	1 NF + 4 F	1 NF + ≥ 5 F
n	28,982	5,648	1,242	333	113	10,713	6,384	2,788	724	157
Mean	9.8	6.46	4.67	3.73	2.48	9.12	9.33	9.38	9.61	9.34
SD	4.78	3.85	3.07	2.75	2.06	4.78	4.83	4.93	5.03	5
Median	9	6	4	3	2	8	8	8	8.5	8
Q1, Q3	6, 14	4, 8	3, 6	2, 5	1, 4	6, 13	6, 13	5, 14	6, 14	5, 14
Min, Max	0, 19	0, 19	0, 18	0, 18	0, 16	0, 19	0, 19	0, 19	0, 19	0, 18

SD = Standard Deviation; Q1, Q3 = first and third quartiles; NF = Non-Fastidious; F = Fastidious
